# Obesity and 25(OH)D Serum Concentration Are More Important than Vitamin D Intake for Changes in Nutritional Status Indicators: A Population-Based Longitudinal Study in a State Capital City in Southern Brazil

**DOI:** 10.3390/nu11102366

**Published:** 2019-10-04

**Authors:** Francieli Cembranel, Eleonora d’Orsi, Katia Jakovljevic Pudla Wagner, Marui Weber Corseuil Giehl, Yara Maria Franco Moreno, David Alejandro González-Chica

**Affiliations:** 1 Department of Nutrition, Federal University of Santa Catarina, Trindade University Campus, Florianópolis, Santa Catarina 88040-370, Brazil; yara.moreno@ufsc.br; 2 Department of Public Health, Federal University of Santa Catarina, Trindade University Campus, Florianópolis, Santa Catarina 88040-370, Brazil; eleonora.dorsi@ufsc.br; 3 The Bernard Lown Scholars in Cardiovascular Health Program, Department of Global Health and Population, Harvard T.H. Chan School of Public Health, 665 Huntington Avenue, Building 1, Room 1210, Boston, MA 02115, USA; 4 Center for Rural Sciences, Bioscience and Unique Health Coordination, Federal University of Santa Catarina, Curitibanos University Campus, Curitibanos, Santa Catarina 89520-000, Brazil; katia.wagner@ufsc.br; 5 Health Sciences Department, Federal University of Santa Catarina, Araranguá University Campus, Araranguá, Santa Catarina 88906-072, Brazil; mwcorseuil@gmail.com; 6 Discipline of General Practice, Adelaide Medical School, Room 813, Level 8, Hughes Building, North Terrace Campus, University of Adelaide, Adelaide, SA 5005, Australia; david.gonzalez@adelaide.edu.au

**Keywords:** dietary vitamin D intake, 25(OH)D serum concentration, body mass index, weight, waist circumference, prospective study

## Abstract

Our objective was to investigate the relationship between dietary vitamin D intake and serum concentration of vitamin D (25(OH)D) on changes in body weight, waist circumference (WC), and body mass index (BMI), and to determine if this relationship changes between obese and non-obese individuals at baseline and those who have or do not have 25(OH)D deficiency. This was a prospective study with a sample of 572 individuals aged 25–65 years, who were participants in the cohort study EpiFloripa Adults. Changes in weight (in kg), BMI, and WC between 2012 and 2014 were evaluated as outcomes. The main exposure was the dietary intake of vitamin D (2012), and the 25(OH)D serum concentration was secondary. When the analyses were stratified by the presence of obesity in the baseline, among obese individuals it was observed that those in the extreme categories of vitamin D intake had an average gain of 3.0 kg in weight, 0.9 kg/m^2^ in BMI, and 1.7–2.7 cm in WC. When 25(OH)D serum concentration were incorporated into the analyses, it was observed that non-obese subjects not having 25(OH)D deficiency had a mean reduction of 2.3 cm in WC. In conclusion, the increases in body weight, BMI, and WC were higher over time in obese patients with deficient 25(OH)D serum concentration, regardless of dietary vitamin D intake.

## 1. Introduction

The progressive increase in the prevalence of obesity since the 1980s has been considered a global public health problem. The World Health Organization estimated the obesity prevalence to be 13% of adults over 18 years for the year 2018 worldwide [1]. The severity of obesity is due in large part to its relation with the occurrence of chronic non-transmissible diseases such as cardiovascular diseases, hypertension, type 2 diabetes mellitus, and various types of cancer. Together, these diseases account for a significant percentage of deaths in the world, ranging from 37% in low-income countries to 78% in middle-income countries, and 88% in high-income countries [1,2].

In addition to traditional risk factors such as physical inactivity, excessive calorie intake, and created environmental factors [2], longitudinal studies in the past decade have emphasized vitamin D deficiency as one of the possible determinants of excessive weight gain and obesity [3,4]. 

Vitamin D is a fat-soluble vitamin obtained by both diet (in smaller amounts) and by sun exposure (main source) [5,6]. It is traditionally recognized for its role in calcium and phosphorus homeostasis in bone tissue [6]. Longitudinal studies in the past decade have shown an inverse relationship between serum concentration of this vitamin (also referred to as 25(OH)D, the major vitamin D metabolite in blood) and body adiposity [3,4]. In Norway, among participants of The HUNT Study, Mai et al. [4] identified that 25(OH)D serum concentrations below 50.0 nmol/L were associated with a significantly increased odds ratio for obesity incidence during follow-up (adjusted OR = 1.73, 95% CI 1.24; 2.41). In northeast Germany and Denmark, through a cohort study, Hannemann et al. [3] identified that 25(OH)D deficiency was associated with the highest quintile of waist circumference. Regarding intake, despite the absence of longitudinal studies, a cross-sectional population-based study with a sample of adults from a city in southern Brazil identified an inverse association between low dietary vitamin D intake and elevated waist circumference values (β −0.69; 95% CI −1.32; −0.06) [7]. 

According to the literature, this relationship is due to the fact that when the 25(OH)D serum concentration lowers, it promotes fat deposition inside the adipocytes, thus favoring weight gain [8]. On the other hand, Vimaleswaran et al. [9] additionally demonstrated that increases in body weight may also lead to a reduction in 25(OH)D serum concentration. Considering that specific data in Brazil indicate that 25(OH)D deficiency may reach more than half of the adult population [10], that vitamin intake presents a dietary inadequacy prevalence of 98.6% [11], and that obesity already affects more than one-fifth of the Brazilian population [12], it is important to also study this relationship among adults in the country.

Thus, this study aimed to investigate the relationship between vitamin D intake and 25(OH)D serum concentration in changes in body weight, waist circumference (WC), and body mass index (BMI) among adults in the city of Florianópolis (the capital city of Santa Catarina state in southern Brazil) based on a prospective cohort study, and to determine if this relationship changes between obese and non-obese individuals at baseline and those who have or do not have 25(OH)D deficiency.

## 2. Materials and Method

### 2.1. Population

This is a prospective study with data from the EpiFloripa Adults study. The cohort study EpiFloripa Adults began in 2009 (baseline) to examine living and health conditions in a representative sample of adults from the city of Florianópolis, the capital city of Santa Catarina state, Southern Brazil. In 2009, Florianópolis had a population of 408,161 inhabitants (60% of which were adults) [13]. Florianópolis is recognized as the city with the third-highest Human Development Index (HDI-M) in Brazil (0.847), and has a low percentage of illiteracy (2.1%) compared to other cities in the country [14].

### 2.2. Sample and Sampling

The sample size of the EpiFloripa Adults cohort study at baseline (in 2009) was calculated considering the following parameters: reference population of 249,530 adults aged 20 to 59 years; confidence level of 95%; prevalence of 50% for unknown outcomes; sample error of 3.5%; design effect of 2.0 (due to cluster sampling); percentage of losses of 10%; plus an additional 15% for adjusting for confounding factors, thereby generating an estimated sample of 2016 adults.

Participant selection was carried out by two-stage clusters: in the first stage, 63/420 census tracts were systematically selected according to the average monthly income of the head of the family (R$192.80 to R$13,209.50; equivalent to US$327.76 to US$22,456.15) and in the second stage, households were selected, 18 in each sector (1134 households, average of 1.7 adults per household). All residents aged 20 to 59 in the selected households were considered eligible. We did not include individuals in the sample who were bedridden, amputated, or with mental problems that interfered in understanding the study questions and/or in anthropometric measurements. Women who were pregnant or who had given birth in the six months prior to the survey were not submitted to anthropometric measurements. Individuals who were not found after four attempts to visit, including one on the weekend and another at night, were not included in the sample. Thus, 1720 adults were effectively interviewed in 2009.

**Figure 1:**
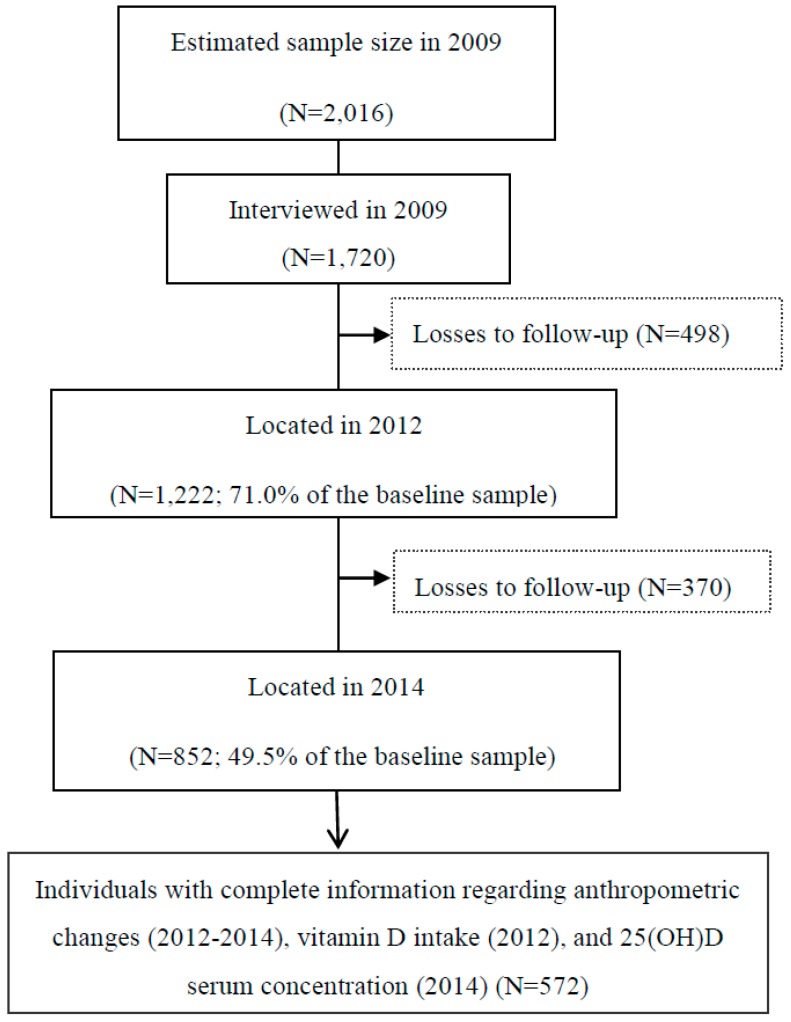
Flowchart of the follow-ups (2009–2012–2014) in the EpiFloripa Adults cohort study. Florianópolis, Santa Catarina, Brazil.

The second and third waves of the study were performed in 2012 and 2014, when 1222 and 852 participants of the original cohort were located (Figure 1).

### 2.3. Data Collection

Interviews were conducted at the participants’ homes in 2009 and in 2012, and the data were recorded in personal digital assistant (PDA) devices.

In 2014, participants were evaluated at the facilities of the Federal University of Santa Catarina (UFSC), and data were recorded on Samsung Galaxy Tab 370^®^ tablets using the Droid Survey^®^ application. The blood samples obtained in this wave were collected after a period of eight hours of fasting and stored at a temperature of −80 °C. The analyses were performed at the Clinical Analysis Laboratory of the UFSC University Hospital.

For quality control, the questionnaires were pre-tested, the equipment was calibrated, and the interviewers were trained and standardized to obtain the anthropometric measurements for all the study waves. Recommended procedures were also followed for the blood sample collection, storage and dosing [15].

### 2.4. Study Variables

#### 2.4.1. Outcomes

The changes in three nutritional status indicators between the years 2012 and 2014 were considered as outcomes: (1) change in weight (in kg); (2) change in BMI (in kg/m², BMI estimated from the equation weight/height^2^); and (3) change in WC (in cm).

The anthropometric measures used to generate these changing variables between 2012 and 2014 were obtained following the standard procedure described in the literature [16]. Body weight (in kg) was measured using a portable digital scale (HCM 5110 Gama Italy Professional^®^, São Paulo, Brazil) with a capacity of 150 kg and a sensitivity of 100 g. The height (in cm) was collected using a portable stadiometer with a capacity of 250 cm and a precision of 1 mm. WC (in cm) was measured in the more narrow trunk region, or at the midpoint between the last rib and the upper border of the iliac crest when the more narrow trunk region was not apparent [17], using an inelastic tape measure (Sanny^®^, São Bernardo do Campo, São Paulo, Brazil) of 160 cm in length and a precision of 1 mm. Thus, differences in weight, BMI, and WC were estimated between the years 2012 and 2014 based on this information.

#### 2.4.2. Main Exposure

Main exposure was dietary vitamin D intake. Data on vitamin D intake (in μg) were obtained in 2012 by means of two 24-h recall questionnaires. The first 24-h recall questionnaire was answered by the entire sample and the second by a subsample of 40% of the respondents, following a methodology described in the literature [18]. This methodology was adopted to adjust this variable by intra-individual and inter-individual variability, as recommended by the National Research Council and the Institute of Medicine [19,20,21], and by energy intake [22]. Additional details about these methodological procedures can be reviewed in a previous publication [7]. In the present study, vitamin D intake was analyzed as a continuous and categorical variable (ingestion tertiles).

#### 2.4.3. Secondary Exposure

Serum concentrations of 25(OH)D were obtained from the participants’ blood samples from 2014. Microparticle chemiluminescent method was used to determine serum concentrations [15]. This variable was analyzed as continuous (ng/mL) and dichotomous (deficiency: 25(OH)D ≤ 20.0 ng/mL) [23].

#### 2.4.4. Co-Variables

In agreement with the literature [3,4,7,24], the following adjustment variables were included in the performed analyses: sex (female, male); age in full years; skin color (white, other color); relationship status (married/living with partner, single/separated/divorced/widowed); schooling in full years of study; family income per capita in reais (R$); the use of vitamin supplements; anthropometric measures (weight, height, and WC) and BMI of 2012; and 25(OH)D serum concentration.

### 2.5. Data Analysis

The data are presented in percentage (%) to describe the categorical variables, and the data are expressed as means with their respective standard deviations and in medians and interquartile range to describe the continuous variables. The differences in weight, BMI, and WC between the years 2012 and 2014 are presented as deltas.

Linear regression was used in both the crude and adjusted analyses to estimate the association of vitamin D intake (2012) and 25(OH)D serum (2014) with the changing variables (weight, BMI, and WC) between 2012 and 2014. The possible confounding factors were included together in the adjusted analyses. Anthropometric measures (weight, height, WC) and BMI (from 2012) were also included as adjustment variables. The results are presented as regression coefficients (β) with their respective confidence intervals (95% CI). Determination coefficients (*R*^2^) were also calculated as a measure of model explanation.

In addition, to test whether dietary vitamin D intake influenced weight gain/adiposity differently between obese and non-obese individuals at baseline, the association between vitamin D intake (in 2012) and changing variables (weight, BMI, and WC) between 2012 and 2014 were stratified by the presence of obesity at baseline and by vitamin D intake in tertiles. Serum concentrations of 25(OH)D (deficiency < 20 ng/mL) were also tested in the analyses as a possible additional source of heterogeneity in order to verify whether obese and non-obese subjects at baseline with adequate 25(OH)D serum concentration had lower weight gain/adiposity than those with 25(OH)D deficiency, independent of the intake tertiles. Multiplicative terms among these variables were incorporated into the regression models, considering *p*-values ≤ 0.10 as evidence of effect modification [25].

All analyses were conducted in Stata software version 14.0 (StataCorp^®^, Bryan, TX, USA), using sample weights (which were determined considering the selection probability of participants in 2009 and location in 2014 in each census sector).

## 3. Results

In the analyses of the present study, data were used only from the participants of the EpiFloripa Adults cohort study with complete information for all variables of interest in the waves of 2012 and 2014, resulting in *N* = 572 individuals. However, it is unlikely that the reduced sample size would have biased the results, considering that the sociodemographic characteristics remained at a similar percentage between baseline and the cohort follow-up in 2014 (Table 1).

In 2012, the mean weight among men was 78.0 kg (SD ± 15.2), mean BMI was 26.4 kg/m^2^ (SD ± 4.6), and mean WC was 91.5 cm (SD ± 12.8). The corresponding values among women were 66.9 kg (SD ± 13.7), 26.2 kg/m^2^ (SD ± 5.2), and 82.9 cm (SD ± 13.2). Regarding the changing variables (weight, BMI, and WC) between 2012 and 2014, the results revealed small differences between men and women (∆weight_men_ = 0.4 ± 3.7 kg and ∆weight_women_ = 0.3 ± 5.7 kg; ∆BMI_men_ = −0.1 ± 1.2 kg/m^2^ and ∆BMI_women_ = −0.04 ± 2.2 kg/m^2^; ∆WC_men_ = −1.4 ± 4.9 cm and ∆WC_women_ = −1.6 ± 5.5 cm; *p* > 0.05 for all cases) (data not presented in tables). 

The average dietary vitamin D intake in 2012 was 4.47 μg/day (SD ± 0.37). 25(OH)D serum concentration in 2014 were 23.2 ng/mL (SD ± 5.9). The correlation between vitamin D intake in 2012 and 25(OH)D serum concentration in 2014 was weak (*r* = 0.1, 95% CI −0.02, 2.82) (data not presented in tables).

The results of the association analysis between vitamin D intake (2012) and the 25(OH)D serum concentration (2014) with the variables (weight, BMI, and WC) between 2012 and 2014 are presented in Table 2. Dietary vitamin D intake showed no association with any of the changing variables in eit ikki her the crude or adjusted analyses. Serum concentrations of 25(OH)D were inversely associated with changes in the three nutritional status indicators in the adjusted analysis. However, the highest *R*^2^ was 1% for the association between the 25(OH)D serum concentration and the change in WC. There was no evidence of effect modification in associations according to sex or age (*p*-value > 0.10 in all cases).

**Figure 2:**
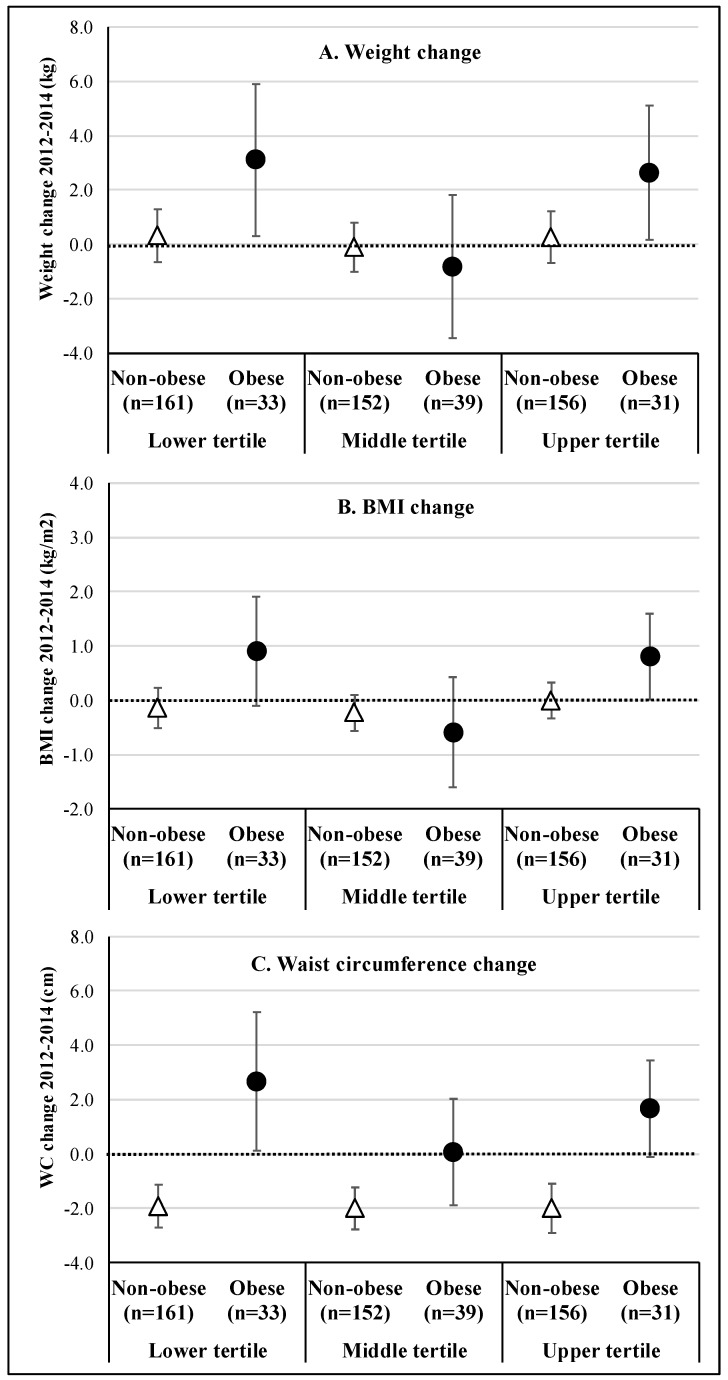
Adjusted ^a^ association between vitamin D intake (2012) in tertiles and change variables (weight (**A**), BMI (**B**), and WC (**C**)) between 2012 and 2014, stratified by the presence of obesity (BMI ≥ 30.0 kg/m^2^) at baseline. ^a^ = Adjusted for sociodemographic characteristics (sex, age, educational level, skin color, relationship status, family income per capita), vitamin supplementation, anthropometric measurement (height, weight, WC) and BMI at the beginning of the period (2012). Vertical lines represent the 95% confidence intervals.

The results of the association analysis between vitamin D intake (in 2012) in tertiles and the changing variables (weight, BMI, and WC) between 2012 and 2014, stratified by the presence of obesity at baseline, are presented in Figure 2. Among non-obese individuals, both weight and BMI remained stable between 2012 and 2014 (Figure 2A,B, respectively), while WC had a mean reduction of −2.0 cm (Figure 2C), regardless of vitamin D intake tertile. Among obese individuals, a U-ratio was observed between vitamin D intake and changes in the three outcomes, with individuals in the extreme categories of vitamin intake (lower and higher tertile, respectively) presenting an approximate mean gain of 3.0 kg in body weight, 0.9 kg/m^2^ in BMI, and 1.7–2.7 cm in WC.

**Figure 3:**
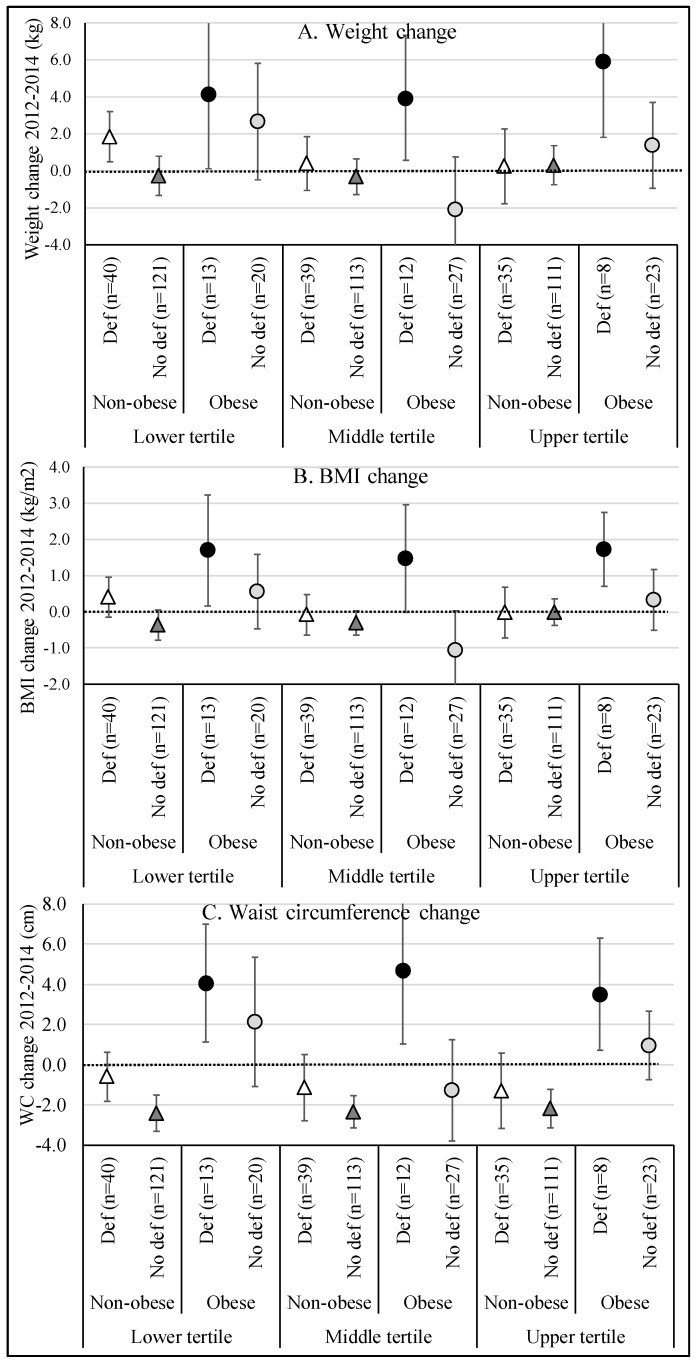
Adjusted ^a^ association between vitamin D intake (2012) in tertiles and change variables (weight (**A**), BMI (**B**), and WC (**C**)) between 2012 and 2014, stratified by the presence of obesity (BMI ≥ 30.0 kg/m^2^) in the baseline and the 25(OH)D serum concentration (2014). ^a^ = Adjusted for sociodemographic characteristics (sex, age, educational level, skin color, relationship status, family income per capita), vitamin supplementation, anthropometric measurement (height, weight, WC) and BMI at the beginning of the period (2012). Vertical lines represent the 95% confidence intervals. 25(OH)D deficiency = 25(OH)D serum ≤ 20 ng/mL.

When 25(OH)D serum concentrations were incorporated into the analyses (deficiency: 25(OH)D serum ≤ 20 ng/mL) as an additional source of heterogeneity, a change in the association pattern was observed (*p*-values for interaction, 0.064 for weight change, 0.043 for BMI change, and 0.529 for WC change) (Figure 3). The weight between 2012 and 2014 remained stable between non-obese individuals (baseline) and no 25(OH)D deficiency, regardless of the vitamin D intake tertile (Figure 3A), but these results were not significant. On the other hand, obese individuals who had 25(OH)D deficiency presented a weight gain greater than and equal to 3.9 kg in the first and second ingestion tertiles, and a gain of almost 6.0 kg in the third intake tertile. Moreover, the relationship among the obese subjects with no 25(OH)D deficiency was “U”, but these results were not significant. A similar pattern was observed in relation to the change in BMI between 2012 and 2014 (Figure 3B). Regarding the change in WC (Figure 3C), regardless of the vitamin D intake tertile, non-obese individuals with no 25(OH)D deficiency had a mean reduction of 2.3 cm in WC, while obese individuals who had 25(OH)D deficiency showed an average increase of 3.5 to 4.7 cm in the WC between 2012 and 2014 (significant results).

When multiplicative terms were added to the final regression models (between vitamin D intake and 25(OH)D serum concentration), there was a 36% increase in variability explained by the models for weight change and BMI (*R*^2^ increased from 12.9% to 17.5%, and from 12.8% to 17.4%, respectively), and of 9.0% for the change in WC (*R*^2^ increased from 31.6% to 34.4%).

## 4. Discussion

To date, this study is characterized as the first population-based prospective study investigating the effect of vitamin D intake and 25(OH)D serum concentration on changes in three nutritional status indicators (weight, BMI, and WC) among adults from a city located in a middle-income country. Two main findings can be highlighted in this investigation: first, increases in BMI and WC over a two-year period were higher among obese (baseline) and 25(OH)D-deficient individuals; second, a significant reduction in WC was observed among non-obese patients (at baseline) and those with no 25(OH)D deficiency. It is noteworthy that both results were independent of dietary vitamin D intake.

These findings are in line with the scientific literature [5,6], which also shows that deficient 25(OH)D serum concentrations are more important for changes related to weight gain/adiposity over time than dietary vitamin D intake, especially in individuals with some degree of pre-existing obesity. This is because very few foods naturally contain vitamin D. According to Holick (2004) [26] and Ashwell (2010) [27], less than 10% of the human requirement for vitamin D is taken from the diet, whereas more than 90.0% of the serum concentration of 25(OH)D depends on regular exposure to the sun. However, Pourshahidi et al. [6] emphasize that obese individuals could cover more of their body with clothing than non-obese individuals, or even participate less in outdoor activities, thereby reducing sun exposure and limiting the endogenous production of cholecalciferol in the skin. In addition, these same authors [6] still draw attention to the fact that vitamin D is a fat-soluble vitamin. Thus, in obese individuals, it may be sequestered by adipose tissue, may be affected by volumetric dilution, or negative feedback mechanisms may occur by increasing circulating 1,25-dihydroxyvitamin D3 (calcitriol) [6]. Therefore, considering that in 2012 the sample in question had prevalences of general and abdominal obesity of 20.2% (95% CI 15.0%; 25.8%) and 28.5% (95% CI 23.8%; 33.5%), respectively, a prevalence of inadequacy in dietary vitamin D intake greater than 99% [7], and that Florianópolis is the third-coldest capital of Brazil (favoring the lower sun exposure throughout the year), these factors are consistent with the evidence that the previous nutritional status of obesity was a more important effect modifier in the tested associations, as well as the serum concentration of 25(OH)D, than diet vitamin D. 

However, longitudinal results differing from those of this study are observed in the literature. For example, a prospective study in the United States of 1081 American adults of African and Hispanic origin found that 25(OH)D serum concentrations were inversely associated with BMI only at baseline (*p* < 0.001 for Hispanics, and *p* = 0.002 for African Americans). However, no significant association was observed between 25(OH)D serum concentrations and changes in BMI after follow-up (5.3 years)—a finding that may be related to the fact that the results of this cohort were not stratified according to the presence of obesity at baseline [24]. A cohort study in Norway [4] with 2165 participants in The HUNT Study found no significant results in assessing the effect of 25(OH)D serum concentration between obese (baseline) subjects and changes in body weight, BMI, or WC at end of the study (11 years of follow-up). In this study [4], 25(OH)D serum concentrations lower than 50.0 nmol/L were only associated with a significantly increased incidence of obesity during follow-up for subjects without the presence of obesity at baseline (adjusted OR 1.73, 95% CI 1.24; 2.41).

As can be seen, the evidence supporting the association between 25(OH)D serum concentration and the changes in nutritional status indicators from longitudinal studies are limited and inconsistent. One possible explanation for this may be related to the fact that anthropometric adiposity indicators such as weight, BMI, and WC are weaker than direct adiposity measures such as visceral/subcutaneous fat [24]. This hypothesis can be confirmed by analyzing the results of another cohort study with adults in Brazil, for example, which identified an important association between 25(OH)D serum concentration lower than 20 ng/mL with visceral adipose tissue (OR 2.00; 95% CI 1.41; 2.86) and subcutaneous adipose tissue (OR 1.58, 95% CI 1.11; 2.24) [28].

As limitations of this study, we emphasize that approximately only one-third of the original cohort was interviewed in 2014. However, it is unlikely that this reduction in sample size would have biased the results, considering that the sociodemographic characteristics of the participants remained similar in the three study waves. Another limitation of this study is the use of 25(OH)D serum concentration obtained from the 2014 wave. Even considering that vitamin D serum concentrations remain stable in the organism for a certain period of time [9], we suggest that new longitudinal studies should be performed using 25(OH)D serum concentrations collected at baseline. In addition, we still believe that this biological marker is unlikely to have been affected by some type of supplementation, since less than 5.0% of the sample was consuming some kind of vitamin supplement (and all results were adjusted for this covariate). In addition, two systematic reviews published in 2015 [29,30] point out that vitamin D supplementation does not affect body weight or BMI changes, even among obese adults. Regarding the strengths of the study, we highlight that this research was carried out with a representative sample of the adults from a city located in a middle-income country, in addition to the methodological care adopted in all the study waves, from data collection up until the analyses of the study.

## 5. Conclusions

Therefore, it is possible to conclude that obese individuals with deficient 25(OH)D serum concentration are more likely to experience gains in weight, BMI, and WC over time, regardless of dietary vitamin D intake. Considering that Brazil is facing a rapid nutritional transition process, the results of this study provide important evidence that may contribute to controlling obesity in the country.

## Figures and Tables

**Table 1:** Comparison of the sample characteristics between baseline (2009) and the follow-up in 2014 ^a^. EpiFloripa Adults Cohort study, Florianópolis, Santa Catarina, Brazil.

Characteristics	Included in 2009 (*N* = 1720)	Included in 2014 (*N* = 572)
Sex (females)—%	57.6	59.8
Age (years)—Mean ± SD	39.4 ± 11.4	39.8 ± 10.9
Skin color (white)—%	91.2	93.2
Relationship status (married or living with a partner)—%	66.9	68.7
Education level (years)—Mean ± SD	12.0 ± 4.7	12.4 ± 4.6
Family income (per capita, in R$)—Median (*p*25–*p*75)	900 (500–750)	1000 (533–1750)

SD = standard deviation; R$—Brazilian currency (US$1.00 equivalent to R$1.70, in 2009); *p*25–*p*75 = interquartile range. ^a^ = Individuals with complete information regarding measurement changes (2012–2014), vitamin D intake (2012) and 25(OH)D serum concentration (2014).

**Table 2:** Crude and adjusted association between vitamin D intake (2012) and 25(OH)D serum concentration (2014) with the changing variables (weight, body mass index (BMI), and waist circumference (WC)) between 2012 and 2014. EpiFloripa Adults Cohort study, Florianópolis, Santa Catarina, Brazil.

	Weight Change 2012–2014 (kg)		BMI Change 2012–2014 (kg/m^2^)		WC Change 2012–2014 (cm)	
	β (95% CI)	*p*	β (95% CI)	*p*	β (95% CI)	*p*
Vitamin D intakein 2012 (μg)						
Crude ^a^	−0.80 (−1.97; 0.38)	0.179	−0.19 (−0.61; 0.24)	0.386	−1.08 (−2.69; 0.54)	0.332
Adjusted ^b^	−0.10 (−1.13; 0.94)	0.853	0.12 (−0.28; 0.52)	0.544	−0.52 (−1.75; 0.71)	0.405
25(OH)D (ng/mL) in 2014						
Crude ^c^	−0.06 (−0.12; 0.01)	0.072	−0.02 (−0.04; 0.00)	0.088	−0.10 (−0.18; −0.01)	0.024
Adjusted ^d^	−0.07 (−0.12; −0.02)	0.006	−0.03 (−0.04; −0.01)	0.007	−0.12 (−0.19; −0.06)	0.001

β: regression coefficient indicating the average anthropometric change for each increase of one unit of vitamin D intake or 25(OH)D serum concentration; 95% CI: 95% confidence interval; ^a^ = *R*^2^ values for the association between vitamin D intake and weight change = 0.4%, BMI change = 0.2%, and WC change = 0.6%. ^b^ = Adjusted for sociodemographic characteristics (sex, age, educational level, skin color, relationship status, family income per capita), vitamin supplementation, anthropometric measurement (height, weight, WC) and BMI at the beginning of the period (2012). ^c^ = *R*^2^ values for the association between 25(OH)D serum concentration and weight change = 0.5%, BMI change = 0.4%, and WC change = 1.2%. ^d^ = Adjusted for ^b^ + vitamin D intake in 2012.
